# Democracy and the Hospitals

**Published:** 1893-12-16

**Authors:** 


### Democracy and the Hospitals.

Hospitals in the United Kingdom are fortunately democratic institutions. To attempt even to sum up the benefits conferred by hospitals upon the democracy would require a small volume; and the writer proposes instead to inquire in what manner and to what extent democracy is endeavouring to repay the accumulated debt of centuries, and to ascertain how far this new power, the democracy, is alive to the obligations and responsibilities under which hospitals have placed it.

Taking a corner of the country covered with hives of industry, and teeming with a virile, active democracy, five chief hospitals are found; and the ceaseless activity of the industrial centres of the West Riding of Yorkshire is alone surpassed by the continuous ministrations of the hospitals in its midst. The chief of these are the Leeds, Bradford, Sheffield, Huddersfield, and Halifax Infirmaries. The last reports of these institutions show that from workpeople's subscriptions, from co-operative societies, friendly societies, and out-door fêtes, the Leeds Infirmary received £6,628, or more than a fourth of its whole income; Bradford, £3,442, or nearly a third; Sheffield, £2,695, or more than a fourth; Huddersfield, £1,665, or nearly a fourth; and Halifax, £2,496, or more than one-third. Whilst the total income of these infirmaries from these democratic sources was £16,926, the other annual subscriptions amounted to £12,745. In other words the democracy gave to these five hospitals £4,181 more than the annual subscribers, in the year for which the last reports were issued. In every instance, except that of Sheffield, the sums raised by the democracy exceed the total given by the annual subscribers. The money raised by Hospital Sunday does not enter into either of the calculations.

How and whence are these large sums raised? In Leeds and Bradford there is a highly developed organisation for the encouragement of democratic subscriptions, and it is in these places that the best results are obtained. In each place a committee quite independent of the infirmary, and over whose working the infirmary has no control, undertakes the work. The committees are composed of working men who further the interests of the local infirmary among their comrades, as well as organise the mode of collection. At Bradford the committee is known as the Joint Hospital Committee, and to realise the success of its labours it is only necessary to know that since 1864 it has divided the large sum of £33,018 between the three medical charities of the town. The greater part of the money has been raised by an annual out-door fête or gala. Whilst the Leeds committee can point with no less pride to the £6,377 raised by its instrumentality in 1892 by workpeople's subscriptions alone, on behalf of the three medical charities of the City of Leeds. Without the committee—without the organisation—it is doubtful if a fourth part of these sums would have found its way into the hospital coffers. In striking contrast with the workpeople's contributions of the other four infirmaries is the sum raised at Huddersfield, where, with a larger total income than its neighbour, Halifax, can boast, it raises £987 from workpeople, as compared with £2,022 by Halifax; Bradford and Sheffield being not far behind with £1,895 and £1,714 respectively. Though a season of bad trade may have something to do with the smaller total raised at Huddersfield, the absence of any organisation must bear the brunt of the blame. No hospital secretary, however able and zealous, can cultivate the ground and garner the rich, ripe harvest of the workpeople's subscriptions, unless he have the organised help of those in touch with the workpeople. With clerical and office work, with looking up old subscribers and seeking new ones, with attending to the wants of boards and committees, and superintending the thousand and one details which arise in the working of the hospital, he has his hands full enough. Moreover, there is no better organiser, no more sincere admirer of hospitals, no harder worker in any cause he engages than the intelligent working man; whilst the mass of his comrades listen and respond more readily to the appeals of one of themselves than they will to the fine words and, to them perhaps, stilted phrases of those with whom they can feel little in common.

To show what may be done by organisation, the writer may cite a small outlying village, where the working men annually hold what they call "a demonstration" on behalf of one of the infirmaries mentioned in this paper. For weeks, and even months, before the event, the committee of working men is busy making its preparations; and when the day arrives a procession starts from the village into the town; and after perambulating the principal streets returns to the field where the demonstration is held. To encourage the sale of tickets before the day of the demonstration, the tickets sold are numbered, and prizes are given to the holders of certain tickets, and a prize is given to the seller of the largest number of tickets before the day, the price of a ticket purchased before the day being fourpence, and on the day of the demonstration sixpence. Fantastically garbed collectors, mounted and on foot, accompany the procession, and the collector of the largest sum from the spectators on the line of route receives a prize. And what are the results?

Besides providing a half-day's recreation for the people, and reminding them of the needs of the infirmary, this little village has sent £480 to the infirmary funds during the last five years. The chairman of the committee has been elected a life-governor of the infirmary—a recognition of the help his committee has given, which has been much appreciated; and he is also one of the working-men members of the board of management of the infirmary, and from personal observation knows something not only of the work done by the institution, but of the care which is used in the disbursal of the funds so liberally contributed by his fellow-workmen.

The other sources of democratic contributions, with the total at each infirmary, are as follows:—

*Friendly Societies' Demonstrations.*—Bradford, £1,500; Leeds, £418; Huddersfield, £122; Sheffield, £126; and Halifax, £5.

*Benefit, Trade, and Friendly Societies.*—Leeds, £444;

Halifax, £165; Huddersfield, £149; Bradford, £115; and Sheffield, £64.

*Co-operative Societies.*—Huddersfield, £163; Leeds, £113; Bradford, £108; Halifax, £83; and Sheffield, £5 5s.

*Cricket and Football Matches.*—Leeds, £494; Bradford, £333; Huddersfield, £123; Halifax, £90; and Sheffield, £55.

*Outdoor Musical Festivals.*—Leeds, £526; Halifax, £131; Huddersfield, £121; Bradford, £53; and Sheffield, £24.

Such are some of the modes by which democracy supports the hospitals of the West Riding; and there is no more flattering testimony to the value and efficiency of the hospital service than the voluntary and yearly-increasing contributions of that class, which from actual personal knowledge knows the character of the work done in hospitals, as well as, if not better than, anyone else. It is surely not necessary to point out that the democratic field is one which secretaries and boards of management may cultivate with the surety of reaping a bountiful return for all the care they bestow upon it; nor to repeat that the key to unlock the gate opening on this fertile soil is organisation—organisation outside the hospital, beyond the control of, though in constant touch with the hospital management.

